# The prevalence of secondary neoplasms in acromegalic patients: possible preventive and/or protective role of metformin

**DOI:** 10.1007/s10147-021-01895-y

**Published:** 2021-03-13

**Authors:** Denise Costa, Filippo Ceccato, Rosa Lauretta, Valeria Mercuri, Tania D’Amico, Corrado De Vito, Carla Scaroni, Marialuisa Appetecchia, Patrizia Gargiulo

**Affiliations:** 1 Department of Experimental Medicine, Endocrinology-Pituitary Disease, “Sapienza” University of Rome, Rome, Italy; 2 Endocrinology Unit, Department of Medicine DIMED, University-Hospital of Padua, Padua, Italy; 3 Department of Neuroscience DNS, University of Padua, Padua, Italy; 4 Oncological Endocrinology Unit, IRCCS, “Regina Elena” National Cancer Institute, Rome, Italy; 5 Department of Public Health and Infectious Diseases, “Sapienza” University of Rome, Rome, Italy

**Keywords:** Acromegaly, Secondary tumor prevalence, Metformin, Benign neoplasm

## Abstract

**Background:**

Acromegaly is a rare disease due to chronic growth hormone (GH) excess and the consequent increase in insulin-like growth factor-1 (IGF-1) levels. Both GH and IGF-1 play a role in intermediate metabolism affecting glucose homeostasis. The association between hyperinsulinemia/impaired glucose tolerance and an increased risk of cancer has been clarified. Insulin has a mitogenic effect through its interaction with the IGF-1 receptor (IGF-1R) that also binds IGF-1. On the other hand, metformin, an anti-hyperglycemic drug that decreases serum levels of insulin and IGF-1, could have a protective role in the treatment of endocrine tumors.

**Methods:**

A retrospective, observational, multicenter study in 197 acromegalic patients, receiving/not receiving metformin, was performed to assess whether the prevalence of neoplasms might be correlated with insulin resistance and could eventually be modified by metformin treatment.

**Results:**

In general, the occurrence of secondary neoplasia among our patients was significantly (*p*V = 0.035) associated with a positive family history of malignancy and with disease duration; a trend towards significance was observed in patients aged > 50 years. Acromegalic subjects who had undergone surgery showed a lower probability of developing a malignant tumor, whereas a higher prevalence of malignancies was observed in obese patients. No significant statistical difference was found when comparing metformin-treated or -untreated subjects for the presence of a second tumor. More interestingly, a trend towards statistical significance (*p*V = 0.065) was demonstrated in the metformin-treated group for the onset of a benign neoplasm.

**Conclusion:**

Metformin could act directly on tumor cell metabolism and may have an adjuvant role in benign lesion progression.

## Introduction

The over-secretion of growth hormone (GH) characterizes acromegaly, a rare chronic disease with a worldwide prevalence of 40–130 per million, most commonly due to a pituitary tumor, and associated with multiple comorbidities, such as diabetes mellitus (DM), sleep apnea, arthropathy, cardiovascular system disorders, and cancer [1]. Early diagnosis and prompt treatment of acromegaly, aimed at obtaining strict control of excess GH, is the best strategy to limit the development, or reverse the complications, of acromegaly, and to prevent premature mortality [2].

The excess of insulin-like growth factor-1 (IGF-1) in acromegalic patients, ascribed to GH overexpression, seems to be involved in the onset of hyperglycemia (with a prevalence of DM ranging from 12 to 37%); it also promotes insulin resistance by lipolysis, stimulating gluconeogenesis, and blocking the signaling of insulin mediators, such as p85 alpha and insulin receptor substrate (IRS-1) [3].

In recent years, several studies have reported the association between hyperinsulinemia, impaired glucose tolerance (IGT), DM, obesity, and an increased risk of cancer, estimated to be around 20% for breast cancer and 25% for liver and endometrial cancer [4].

The role of glucose and glutamine in supporting the anabolic growth of proliferating cells has been highlighted. Proliferating cells, despite enough oxygen to support aerobic glycolysis, take advantage of glucose to produce greater quantities of lactate, the main source of carbon (Warburg effect) [5]. Similarly, among peripheral tissue chronic outcomes, hyperinsulinemia promotes malignant transformation, through both direct and indirect mechanisms [6]. It is also known that insulin has a mitogenic effect through its interaction with the IGF-1 receptor (IGF-1R), a powerful growth factor with transforming and anti-apoptotic effects [4]. An interesting role is played by the insulin receptor isoform A, normally present during fetal development, which has a particularly high affinity for IGF-2. Indeed, in adults its aberrant expression on proliferating cells promotes tumor growth and resistance to therapies, including those targeting IGF-1R [7]. GH is also implicated in tumor growth, altering regulation of the phosphoinositide-3-kinase–protein kinase B/Akt (PI3K/AKT) pathway, which leads to increased activation of mammalian target of rapamycin (mTOR) [1].

Metformin is a first-choice treatment in overweight patients with insulin resistance and/or type 2 DM [8]. It inhibits complex 1 of the mitochondrial respiratory chain in hepatocytes, skeletal muscle, endothelial cells, pancreatic beta cells and neurons, promoting adenosine triphosphate unbalance, with an increase of intracellular adenosine monophosphate (AMP) levels [9]. In turn, AMP activates AMP-activated protein kinase (AMPK). AMPK monitors energy status and protects cellular functions during energy restriction, inhibits cell proliferation and is considered a tumor suppressor. Specifically, it activates the complex with Tuberous Sclerosis 1–2 (TSC1-TSC2), which regulates, by inhibiting it, mTOR complex 1, which in turn regulates protein translation. It is important to underline the role of metformin in decreasing serum levels of insulin and IGF-1, reducing the stimulus for growth and neoangiogenesis [9]. Furthermore, metformin seems to have an antiangiogenic effect, directly scavenging free radicals and blocking endogenous reactive oxygen species [10].

Despite the above-mentioned mechanisms of metformin, data concerning its role as adjuvant therapy in the treatment of cancer are still under debate [9–15]. The aim of this study was to evaluate whether metformin treatment, in addition to modulating insulin resistance, could interfere with the onset of secondary neoplasms in acromegalic patients, confirming or not its role as an antitumoral adjuvant in these subjects.

## Patients and methods

Acromegalic patients regularly attending three Italian centers for the management of pituitary diseases (Department of Experimental Medicine-Endocrinology—“Sapienza” University of Rome; Oncological Endocrinology Unit, IRCCS—“Regina Elena” National Cancer Institute and UOC Endocrinology; Endocrinology Unit, University of Padua), were enrolled in this retrospective, multicenter study.

Demographic and clinical data were obtained from medical reports. Information about the acromegaly diagnosis, symptoms and signs were collected between 2000 and 2019. Inclusion criterion was the diagnosis of acromegaly by 2014, to ensure a follow-up of at least 5 years. The diagnosis of acromegaly was performed according to the guidelines, which, in patients with elevated or equivocal serum IGF-1 concentrations, recommend confirmation of the diagnosis by finding a lack of suppression of GH to < 0.4 μg/L, following documented hyperglycemia during an oral glucose load (2 h after 75 g of oral glucose) [16]. Biochemical disease control was defined by determining IGF-1, age-related, and GH expressed in ng/mL [16]. Patients with pituitary adenomas with a Ki-67 proliferation index > 3% were also included in the study. These adenomas, resulting from histopathological evaluation in patients treated surgically, have been considered more aggressive forms.

Follow-up was also performed according to international guidelines [1, 17]. A screening colonoscopy was carried out at diagnosis. If the colonoscopy was negative, the patients were screened similarly to the general population, especially if insulin-like growth factor-I (IGF-1) levels were normalized. On the contrary, colonoscopy was performed every 3 years when patients were found with adenomas at first visit or if patients were scarcely responsive or resistant to acromegaly treatments.

All acromegalic patients have undergone standard periodical examinations of thyroid morphology and function, mammography and gynecological examination, abdominal and prostatic ultrasound, based on age, sex, family history, and biochemical control of the disease.

The second neoplasms were diagnosed not only after the diagnosis of acromegaly but also in an interval of time prior to the date of diagnosis (the diagnostic latency period) in which the IGF-1 was probably high and therefore could have had an impact on the development of these neoplasms. The prevalence of a second neoplasm, benign tumor or cancer, was analyzed; association with patient age, sex, malignancy familiarity, duration of disease and symptoms’ latency were also evaluated.

The glycol-metabolic derangement has been stated as DM, IGT, obesity, insulin resistance using current criteria for the enrolled patients [18].

### Statistical analysis

Descriptive analysis was performed using absolute and relative frequencies to describe qualitative variables while mean and standard deviation (SD) were calculated for quantitative variables. A univariate analysis was successively carried out using the following tests: $\chi^{2}$ to compare proportions and Student’s *t*, or the analogous nonparametric Mann–Whitney, to test differences between quantitative variables, as appropriate. A two-tailed *p*-value of 0.05 was considered significant.

The Odds Ratio (OR) and 95% confidence intervals (95% CI) were estimated for each variable using the univariate logistic regression model. The following variables were tested in the model: gender, age, latency of disease signs, duration of disease, HOMA index, presence of obesity, presence of DM, type of pharmacological therapy, radiotherapy, metformin therapy, and surgery. Variables testing significant by univariate analysis were entered into multivariate analysis. A multivariate logistic regression model was developed using stepwise regression (forward selection) to identify variables independently associated with having secondary neoplasm. Enter limit and remove limit were *p* = 0.10 and *p* = 0.15, respectively. IGF-1 values were expressed as a percentage of the upper limit of normal (%ULN), stratified by age groups and subjects were divided into a remission group (IGF-1 ≤ ULN) and an active group (IGF-1 > ULN).

All analyses were performed using Stata 15.1 (Stata Corporation, College Station, Texas, USA, 2017).

## Results

The study enrolled 197 acromegalic patients (75 males, 122 females); clinical and anamnestic data are shown in Table 1. Patients’ median age was 59 years (range 32–83 years) and disease duration 16.8 ± 10 years. Most of the patients (194/197) received treatment for acromegaly; as expected, neurosurgery, radiotherapy, pharmacological therapy, alone or in combination, were the main interventions used to control the pathology. Two patients, who underwent surgery, presented a more aggressive form of pituitary adenomas with a Ki-67 proliferation index > 3% on histopathological evaluation. Neither of them presented a second neoplasm, at the time this report was written.

**Table 1:** Clinical and anamnestic data for all acromegalic patients (*n* = 197)

Variables	All patients *n* (%)
Sex	
M	75 (38.07)
F	122 (61.9)
Age	
Mean (SD)	59 (11)
Duration of disease mean (SD)	16.8 (10)
Diagnostic latency mean (SD)	3.4(5.3)
Initial values	
GH mean (SD)	15.4 (11)
IGF1 mean (SD)	719.4 (289)
Latest values	
GH mean (SD)	1.00 (0,3)
IGF1 mean (SD)	203.6 (70)
Diabetes mellitus	
No	149 (75.6)
Yes	48 (24.3)
Body Mass Index (BMI) mean (SD)	28.05 (4.7)
HOMA-IR Index mean (SD)	4(3)
All secondary neoplasm	131 (66.5)
Secondary neoplasm	
Benign	87 (44.2)
Malignant	44 (22.3)
NT	3 (1.5)
NS	14 (7.1)
NS + DA	2 (1.01)
NS + SSA	44 (22.3)
NS + SSA + PEG	13 (6.6)
NS + SSA + DA	18 (9.1)
NS + SSA + PEG + DA	20 (10.1)
NS + RT	7 (3.5)
NS + RT + SSA	7 (3.5)
NS + RT + PEG + SSA	9 (4.5)
NS + RT + DA + SSA	9 (4.5)
NS + PEG	1 (0.5)
RT	1 (0.5)
RT + SSA	1 (0.5)
SSA	22 (11.1)
SSA + DA	13 (6.6)
SSA + PEG	7 (3.5)
SSA + DA + PEG	3 (1.5)

*DA* dopamine agonists, *NS* neurosurgery, *NT* no treatment, *PEG* pegvisomant, *RT* radiotherapy, *SSA* somatostatin analogs

Data about malignancy familiarity were available for 142/197 patients, 99 of which without any familiarity and 43 with familiarity for cancer of unspecified nature; the prevalence of neoplasia was significantly correlated to a positive family history of malignancy ($\chi^{2}$ = 4.43 *df* = 1; *p*V = 0.035). For disease duration > 15 years ($\chi^{2}$ = 4.08 *df* = 1; *p*V = 0.044) and age > 50 years ($\chi^{2}$ = 3.66 *df* = 1; *p*V = 0.056), a significant trend was also observed.

Second tumor removal surgery was necessary in 129/197 (65%) acromegalic subjects. Histology diagnosed a benign lesion (colon polyposis, multinodular goiter and uterine fibromatosis) in 86/129 (66.6%) and a malignant neoplasia (breast, uterus, kidney, prostate, colon and thyroid cancer) in 43/129 (33.33%) patients.

In the observed case series, 42/197 patients received metformin (1000–3000 mg/day), for at least 5 years for diabetes or insulin resistance (group A), and 155/197 did not receiving metformin (group B).

In Table 2, clinical and anamnestic data for group A (metformin-treated) and group B (not receiving metformin) patients are presented. In group A, 32/42 patients (76.2%) presented neoplasia and, among these, 20/42 (47.6%) had a benign lesion and 12/42 (28.6%) had a malignant neoplasia. In group A, DM was diagnosed in 35/42 (80.9%) patients. In group B, 99/155 (63.9%) presented neoplasia and, among these, 67/155 (43.2%) had a benign lesion and 32/155 (20.6%) had a malignant neoplasia. In group B, 13/155 (8.4%) patients had a diagnosis of DM.

**Table 2:** Association between clinical, anamnestic data and group A vs B

Variables	Group A (42 pts) *n* (%)	Group B (155 pts) *n* (%)	*p* value (*p*V)
Sex			
M	17 (40.5)	60 (38.7)	0.160
F	25 (59.5)	95 (61.3)	0.280
Age			
Mean (SD)	60 (12)	58 (11)	0.056
Duration of disease mean (SD)	19 (10)	16 (10)	0.044
Diagnostic latency mean (SD)	5 (6)	3 (4)	0.284
Initial values			
GH mean (SD)	10.36 (9)	16.81 (12)	0.10
IGF1 mean(SD)	626.25 (284)	748.82 (287)	0.19
Latest values			
GH mean (SD)	0.62 (0.3)	0.99(0.3)	0.86
IGF1 mean (SD)	213.69 (86)	200.7 (66)	0.57
At moment of study			
GH < ULN	42 (100)	155 (100)	1
IGF-1 < ULN	42 (100)	155 (100)	1
Diabetes mellitus			
No	7 (16.7)	142 (91.6)	0.170
Yes	35 (83.3)	13 (8.4)	
Body Mass Index (BMI) mean (SD)	31 (5)	27.1 (4)	0.001
HOMA-IR Index mean (SD)	4.9 (5.3)	3.2 (2.5)	0.248
Secondary neoplasm			
Benign	31 (72.1)	76 (56.3)	0.065
Malignant	12 (27.9)	28 (20.6)	0.339

Comparing groups A and B, no significant statistical difference was observed for the onset of a second tumor ($\chi^{2}$ = 2.92 *df* = 1; *p*V = 0.087).

However, regarding benign neoplasms in acromegalic patients, the comparison between benign neoplasms in group A 31/43 (72.1%) vs group B 76/135 (56.3%) was at the limit of statistical significance ($\chi^{2}$ = 3.39 *df* = 1; *p*V = 0.065).

On the contrary, the analysis of patients with malignant tumors showed no significant statistical difference comparing those treated with metformin and those not receiving metformin ($\chi^{2}$ = 0.91 *df* = 1; *p*V = 0.339).

Moreover, a trend towards significance was found regarding sex (male; $\chi^{2}$ = 3.19 *df* = 1; *p*V = 0.074), age > 50 years ($\chi^{2}$ = 3.35 *df* = 1; *pV* = 0.067), and disease duration > 15 years ($\chi^{2}$ = 3.11 *df* = 1; *pV* = 0.078), in patients with benign tumors, in both groups A and B. No significant correlation was found for malignant tumors in group A and B, matched by sex, age, disease duration, diagnostic latency, or malignancy familiarity.

It is interesting to underline that a higher prevalence of malignancies was observed in obese patients, regardless of treatment with metformin ($\chi^{2}$ = 7.45 *df* = 1; *p*V = 0.006). There was no statistically significant difference between obese diabetic patients and non-diabetic patients. The correlation between HOMA Index and neoplasms was not significant. Acromegalic subjects who underwent surgery showed a lower probability of having malignant tumors compared to those non-surgically treated (OR 0.05; 95% CI 0.01–0.81; *p*V = 0.035). Furthermore, patients treated only with pharmacological therapy, somatostatin analogs (SSA) or pegvisomant (PEG), showed no difference regarding the duration of disease, in relation to the multivariate analysis and when correcting the analysis for target (Table 3).

**Table 3:** Logistic multivariate analysis for the relevant outcome

Variables	All secondary neoplasm	
	OR (CI 95%)	*p* value (*p*V)
SSA	2.6	0.660
DA	4.56	0.337
PEG	0.11	0.2
RXT	3.32	0.357
NS	0.05	0.035

Patient hormonal data are described in Tables 1 and 2.

At the diagnosis of tumor, 62/155 (40%) of subjects in group B and 16/42 (38,09%) in group A had active disease (IGF-1 > ULN) (range 7–493% above ULN). At the moment of the study all subjects were in remission (IGF-1 ≤ ULN) (range 0 to − 70% ULN) for at least 2 years.

Associated pituitary defects, if any, were corrected by adequate replacement therapy.

## Discussion

With the aim of evaluating the impact of metformin treatment on the risk of developing malignancies, the prevalence of neoplasia was calculated in acromegalic patients who have been treated with metformin for at least 5 years. The prevalence of neoplasia was also correlated with clinical-anamnestic parameters. Our study analyzed data from 197 patients with acromegaly.

Based on the mechanism of action of metformin, a significant difference in favor of metformin treatment could be expected.

In general, GH hypersecretion, a condition present in acromegalic patients, chronically engraves regulation of the phosphoinositide-3-kinase–protein kinase B/Akt (PI3K/AKT) pathway, which leads to an increased activation of mammalian target of rapamycin (mTOR) [5]. By increasing intracellular AMP levels [9], metformin induces an increase in AMPK which normally inhibits cell proliferation and is considered a tumor suppressor. Specifically, it activates TSC1-TSC2, which inhibits mTOR complex 1, regulating protein translation [9].

However, in our patients, we observed no statistically significant difference in the prevalence of neoplasms between acromegalic patients treated or not treated with metformin. Indeed, the onset of secondary neoplasm seems to be independent of insulin resistance and metformin treatment.

On the other hand, considering the onset of benign neoplasms, a trend towards statistical significance was observed between patients treated with metformin and those not treated with metformin. All patients in group A were in therapy with metformin for at least 5 years, regardless of the diagnoses of cancer, in relation to a preventive and protective role against malignant transformation of benign neoplasms or metastasis of malignant neoplasms.

According to the literature, the most common benign neoplasms are colon polyposis, multinodular goiter, and fibromatosis [19, 20]. Acromegalic patients aged > 50 years, a longer disease duration (> 15 years), and positive familiarity with neoplasms, seem to be more prone to the development of benign tumors. Moreover, in these patients, due the benign nature of neoplasm, a longer follow-up is achievable, which allows more information to be collected over time.

On the other hand, no correlations with age, sex, disease duration, malignancy history, nor disease duration, were evident in the context of malignant tumors. This may be partly justified because higher proliferating tumors, such as those which are hormone-dependent, escape control mechanisms.

As early as 1963, a study published in Cancer Research emphasized that hormones play an important role in the growth of some breast, prostate and uterine cancers [21]. These three forms of neoplasms constitute more than 34% of the malignant tumors found in our acromegalic patients. However, assessing the prevalence of breast cancer in acromegaly is particularly challenging as it may be looked at only in women. In addition, excess IGF-1 could have different effects depending on menopausal state, and on the levels of other sex hormones, further increasing complexity [22].

Tumor onset appears to be associated with obesity. Obesity-related inflammatory mechanisms can be invoked. It is well known that inflammatory cytokines have tumor-promoting effects, mainly mediated by the activation of nuclear factor 1 B (NF1 B) and signal transducer and activator of transcription 3 (Stat3) signaling pathways, which contribute to angiogenesis and tumor cell changes [23]. In acromegalic patients, these mechanisms, amplified by the action of GH and IGF-1, seem to have a more significant impact on the onset of tumors, compared to insulin resistance.

Growth hormone (GH) has anabolic and mitogenic effects, and insulin-growth factor-1 (IGF-1) is a strong tumor mitogen. IGF-1 serum concentrations are expected to be reduced by drug treatment and, therefore, potentially reducing the risk of tumor development.

Somatostatin analogs are, at present, the most widely used drugs to control acromegaly and systemic effects of GH excess. Octreotide and Lanreotide are two analogs, with comparable binding profile (high affinity for somatostatin receptor subtypes 2 and 5 and a faint affinity for subtype 3) and a similar efficacy in suppressing GH and IGF-1 levels [24, 25].

Indeed, Somatostatin analogues (SSA) are well-established antisecretory drugs that have been used as first-line treatment for symptomatic control in hormonally active neuroendocrine tumours (NET) [26].

Pegvisomant bases its efficacy on blocking the activity of the GH-R, thereby inhibiting the synthesis of IGF-1 and, therefore, potentially reducing the risk of tumor development. Results of studies using human cell lines suggested that the structure of Pegvisomant itself lacks tumorigenic potential [27].

As highlighted by our analysis, no significant differences - were shown between the different types of drug treatment in relation to the onset of second malignancies, correcting the analysis for target too, regardless of duration of disease.

In acromegaly, it has been shown that when excess GH was controlled by pharmacological therapy, the prevalence of malignant neoplasia was higher than that in subjects undergoing neurosurgery [28, 29]. Surgery can be considered a "protective" factor with respect to chemical control of the disease: as expected, our analysis also confirmed this. At the same time, there seems to be no association between pharmacological treatment chosen to control acromegaly and secondary tumor onset.

Our study certainly has limitations. The group of patients treated with metformin is relatively small in relation to the underlying disease. However, since Acromegaly is a rare disease and considering that the prevalence of impaired glucose metabolism ranges from 12 to 37% of patients [3], the sample could be considered adequate. Since the nature of the study is retrospective and observational, it is not intended to set the timing and duration of treatment with Metformin and to evaluate any malignant transformation of benign neoplasms in the long term. At a later time, it may be appropriate to design a prospective study.

## Conclusion

To the best of our knowledge, this is the first study investigating the role of metformin on the onset of secondary tumors in acromegalic patients. Treatment with metformin can be considered to be a protective factor in the onset of benign neoplasms. As already noted, insulin resistance would not appear to be a factor favoring the onset of neoplasia. Therefore, the protective role of metformin would not be attributable to its indirect action as an insulin-sensitizer, but to the direct activation of AMPK and the consequent influence on tumor cell metabolism. On the other hand, less impact was found for metformin on highly proliferating neoplasms, probably due to the complex mechanisms regulating these tumors.

It must be emphasized that surgery alone has a significant effect on reducing the probability of developing malignant neoplasms, provided that it leads to remission of the acromegalic disease. According to our results, whilst recognizing its role in benign lesions, metformin cannot be considered as an adjuvant drug for the treatment of highly proliferative tumors in acromegaly. Further studies, with increased sample size, could provide more evidence on the role of metformin in acromegaly.
